# Multiparameter single-cell proteomic technologies give new insights into the biology of ovarian tumors

**DOI:** 10.1007/s00281-022-00979-9

**Published:** 2023-01-12

**Authors:** Ionut-Gabriel Funingana, Jacob S. Bedia, Ying-Wen Huang, Antonio Delgado Gonzalez, Kenyi Donoso, Veronica D. Gonzalez, James D. Brenton, Alan Ashworth, Wendy J. Fantl

**Affiliations:** 1 Department of Oncology, University of Cambridge, Cambridgeshire, UK; 2 Cancer Research UK Cambridge Institute, University of Cambridge, Cambridge, Cambridgeshire UK; 3 Department of Oncology, Addenbrooke’s Hospital, Cambridge University Hospitals, NHS Foundation Trust, Cambridge, UK; 4 Department of Urology, Stanford University School of Medicine, Stanford, CA 94305 USA; 5 Baxter Laboratory for Stem Cell Biology, Department of Microbiology & Immunology, Stanford University School of Medicine, Stanford, CA 94305 USA; 6 Helen Diller Family Comprehensive Cancer Center, University of California, San Francisco, 1450 Third Street, San Francisco, CA 94158 USA; 7 Stanford Comprehensive Cancer Institute, Stanford, CA 94305 USA; 8 Department of Obstetrics and Gynecology, Stanford University School of Medicine, Stanford, CA 94305 USA

**Keywords:** Single cell, Mass cytometry/CyTOF, Multiplex cellular imaging, Ovarian cancer, Carboplatin resistance, Immune tumor microenvironment, Multimodal biomarkers

## Abstract

High-grade serous ovarian cancer (HGSOC) is the most lethal gynecological malignancy. Its diagnosis at advanced stage compounded with its excessive genomic and cellular heterogeneity make curative treatment challenging. Two critical therapeutic challenges to overcome are carboplatin resistance and lack of response to immunotherapy. Carboplatin resistance results from diverse cell autonomous mechanisms which operate in different combinations within and across tumors. The lack of response to immunotherapy is highly likely to be related to an immunosuppressive HGSOC tumor microenvironment which overrides any clinical benefit. Results from a number of studies, mainly using transcriptomics, indicate that the immune tumor microenvironment (iTME) plays a role in carboplatin response. However, in patients receiving treatment, the exact mechanistic details are unclear. During the past decade, multiplex single-cell proteomic technologies have come to the forefront of biomedical research. Mass cytometry or cytometry by time-of-flight, measures up to 60 parameters in single cells that are in suspension. Multiplex cellular imaging technologies allow simultaneous measurement of up to 60 proteins in single cells with spatial resolution and interrogation of cell–cell interactions. This review suggests that functional interplay between cell autonomous responses to carboplatin and the HGSOC immune tumor microenvironment could be clarified through the application of multiplex single-cell proteomic technologies. We conclude that for better clinical care, multiplex single-cell proteomic technologies could be an integral component of multimodal biomarker development that also includes genomics and radiomics. Collection of matched samples from patients before and on treatment will be critical to the success of these efforts.

## Introduction

**Fig. 1:**
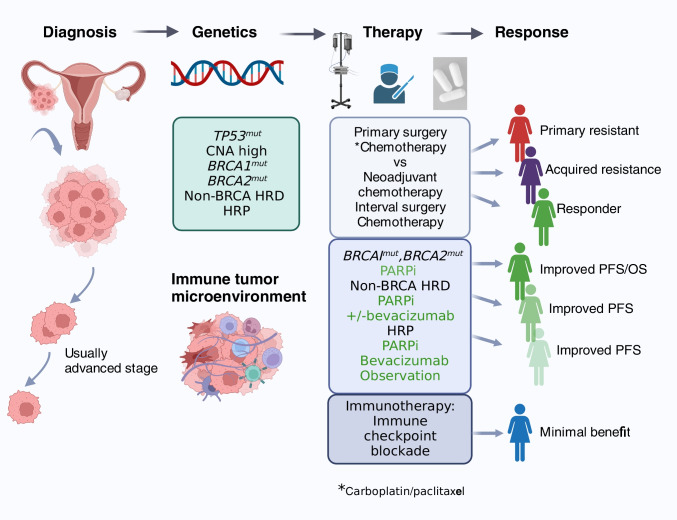
Clinical management of HGSOC: diagnosis, treatment, and response. Diagnosis is usually at advanced stage. A total of 95% cases harbor mutations in *TP53.* A subgroup of tumors harbor mutations in *BRCA1 and BRCA2* (~ 20%) or defects in other genes involved in homologous recombination repair (~ 30%). Patients with newly diagnosed HGSOC are treated either with upfront debulking surgery followed by chemotherapy (usually six cycles of carboplatin plus paclitaxel) or first with three cycles of neoadjuvant chemotherapy, followed by surgery and three additional cycles of chemotherapy. Molecular classification determines response to PARPi shown by shades of green (dark to lighter shades depict long to short PFS). HRD, homologous recombination deficient; HRP, homologous recombination proficient; CNA, copy number alteration; PARPi, poly (ADP ribose) polymerase inhibitor; PFS, progression-free survival; OS, overall survival

High-grade serous ovarian cancer (HGSOC) is the umbrella term for the major sub-type ovarian and fallopian tube carcinoma, as well as primary peritoneal carcinomas. Globally, it is the eighth most common cancer and the most lethal gynecological malignancy with ~ 250,000 new cases diagnosed each year [1–3]. HGSOC is asymptomatic in its early stages and the lack of screening assays for early detection frequently results in patients presenting with advanced-stage disease. This is a major challenge in providing curative treatments and 5-year survival rates remain at < 30% [3, 4] (Fig. 1). Efforts to discover new molecular subtypes and targeted therapies have been hampered by the extreme chromosomal instability (CIN) of HGSOC which results in extensive structural alterations dominated by marked DNA copy number changes [5–7]. Strikingly, the HGSOC genome has few recurrent targetable mutations. The early and ubiquitous development of mutations in *TP53* is permissive for CIN [8]. The most clearly defined genomic subgroups of HGSOC are those with defects in genes involved in homologous recombination (HR) DNA repair estimated to be up to half of HGSOC cases [9]. Of those tumors that are homologous recombination deficient (HRD), a range of 9–30% have been reported with germline or somatic mutation in *BRCA1/2* [10, 11]. Up to 30% of tumors have some degree of HRD due to mutations or epigenetic silencing [12]. Precise definitions are lacking due to the different methodologies used to measure HRD [10, 13]. Forty percent of HGSOC are non-HRD and include subgroups with DNA foldback inversion (FBI) and other signatures of chromosome mis-segregation [6, 14, 15].

For the past 30 years, front-line standard-of-care for women with HGSOC has been debulking surgery combined with carboplatin plus paclitaxel chemotherapy [4, 16, 17]. However, over the past 10 years, use of neoadjuvant chemotherapy has increased [16, 18]. About 6% of tumors are intrinsically resistant to chemotherapy and in 33% patients achieve stable disease by Response Evaluation Criteria in Solid Tumors criteria [19, 20]. Nevertheless, although 60% of tumors respond initially, up to 70–80% will acquire platinum resistance within 3–5 years, during the course of sequential treatments with carboplatin and other agents [21]. The precise mechanisms by which adaptative resistance evolves remain unknown.

Poly (ADP ribose) polymerase inhibitors (PARPi) are the first clinically approved drugs to exploit synthetic lethality within the context of HRD [22, 23]. These drugs have been transformative, especially for HGSOC, as they significantly prolong both progression-free and overall survival [23–27]. While HGSOC tumors that are HRD are sensitive to PARPi, a subgroup of tumors that are apparently homologous recombination proficient (HRP) are sensitive to platinum and respond to PARPi [28]. However, most patients with tumors in either category (HRD or HRP) eventually develop platinum-resistant disease. New curative treatments are thus urgently needed to treat platinum-resistant HGSOC.

Over the past decade, immunotherapy (discussed below) has emerged as a new treatment paradigm in oncology highlighting the critical role of intra-tumoral immune cells [29, 30]. While many malignancies are responsive to immunotherapy (discussed below), to date, this treatment modality has shown minimal clinical benefit in HGSOC suggesting a very immunosuppressive iTME. Whether the latter also plays a significant role in the response to chemotherapy is unclear. We therefore hypothesize that cell-by-cell mapping of the HGSOC iTME is essential to determine the specific cell types conferring resistance to both carboplatin and immunotherapy. We discuss the latest multiparameter single-cell proteomic technologies which are able to identify key intra-tumoral cell phenotypes and their spatial arrangements that may promote therapeutic resistance or response.

## Unraveling the complexity of HGSOC tumors with multi-parameter single-cell proteomic technologies

### Mass cytometry/CyTOF

Tumors, like their normal tissue counterparts, are composed of specialized cells that function together in a coordinated manner. Over the past decade, mass cytometry (cytometry by time-of-flight (CyTOF)) has emerged as a powerful multiplex single-cell proteomic technology that can reveal diverse cell types, especially those in the minority or with subtle differences that would escape detection in bulk analyses. With over sixteen hundred citations in PubMed, the breadth of applications is evident.

CyTOF is an adaptation of flow cytometry in which antibodies are tagged with heavy metal isotopes, rather than fluorophores [31–34]. Unhampered by channel spillover due to overlap of emission spectra, the readout by inductively coupled argon plasma time-of-flight mass spectrometry enables simultaneous measurements of up to 60 parameters per single cell. The mass range for metal detection is between 75 and 209 atomic mass units. However, not all heavy metals within this mass range can be used due to the challenging chemistries required for antibody tagging [35]. In a typical experiment, the majority of metal isotopes are linked to antibodies, but others serve as reagents to assay a variety of key cellular readouts such as DNA intercalation (to register a single cell), cell viability, cell cycle, cellular metabolism, enzymatic activity, hypoxia, and bar coding of samples [36–38]. Critically, the generation of large multi-parameter single-cell datasets necessitated the development of new computational tools which identify cell populations, e.g., clustering approaches to identify cell populations [39] or uniform manifold approximation and projection (UMAP) for single-cell resolution [40].

### Examples of cell populations identified by CyTOF

Initial CyTOF studies enhanced our understanding of the cell types and their activation states within the healthy immune system, permitting aberrant regulation to be identified [41–44]. This has great relevance for cancer patients where the host anti-tumor immune response is key to patient outcome. Importantly, immune cell subpopulations within the peripheral circulation can reflect this response and therefore have been used as a proxy to monitor efficacy of tumor immunotherapy [45–51].

Recently, a CyTOF study of freshly resected, newly diagnosed HGSOC tumors identified a cell subpopulation co-expressing vimentin, cMyc, and HE4, indicative of poor prognosis as well as other subpopulations transitioning between epithelial and mesenchymal compartments (EMT cells) [52]. Follow-up studies can be designed to interrogate these cell types in more detail and the identification of transcripts from single cell (sc)RNA-seq studies can inform the inclusion of additional antibodies into CyTOF panels [53, 54]. Importantly, CyTOF, but not transcriptomic analyses, can measure post-translational modifications, such as phosphorylation, chromosomal modifications, and metabolites providing key insights into intracellular signaling, epigenetics, and metabolomics [38, 41, 55].

### Multiplex imaging

CyTOF necessarily requires single-cell dissociation of tumors thereby destroying all spatial context and incurring loss of components such as stroma that are sensitive to dissociation conditions. Cellular organization is key to understanding tumor progression and response to therapeutic intervention. Conventionally, staining tissue sections with hematoxylin and eosin (H and E) for immunohistochemistry (IHC) provides spatial, morphological, and protein expression data. Despite measurements being restricted to only two to three parameters per section, this approach and the resultant data are used routinely worldwide to diagnose, prognosticate, and inform treatment choices.

In the field of immuno-oncology, where responses to immunotherapeutic agents are not universal, and mechanisms of drug resistance are poorly understood, a far more detailed understanding of cell phenotypes and their spatial interactions is called for. These needs were key motivators for the development of several multiparameter (40–60 parameters per cell) proteomic tissue imaging platforms. As with CyTOF datasets, these platforms spawned the development of new computational tools to analyze the multiparametric spatial data. Each technology utilizes a similar analysis pipeline which includes image alignment and normalization, cell identification, and single-cell signal quantification via a segmentation algorithm, and cell phenotype assignment, usually with an unsupervised clustering algorithm. The end result is an annotated dataset containing each cell’s (*X*, *Y*) coordinates, phenotype, and marker expression profile [56–60]. Each of these imaging platforms enable the cellular architecture of tumor tissue to be characterized in terms of cell–cell interactions and their topographical distribution within a tissue. The datasets are used to derive cellular neighborhoods (CNs) as a standard unit of tissue structure [56, 61]*.* CNs are defined as conserved compositions of cell types that are organized into microstructures within tissue environments [56].

We provide a brief overview of the different imaging platforms; in-depth details are provided in the accompanying citations. Two of the technologies, imaging mass cytometry (IMC) and multiplex ion beam imaging (MIBI) stain samples with heavy metal isotope-tagged antibodies. The readouts are the same as for CyTOF but the antibody clones used may differ. In IMC, a pulsed laser ablates a stained tissue section by rasterizing over a selected region of interest [62]. For MIBI, a primary ion beam composed of atomic ions (i.e., O_2_^+^, Xe^+^) rasterizes over a selected region of interest [63, 64]. For IMC, rasterizing liberates the heavy metal ions bound to antibody for their introduction into the inductively coupled plasma time-of-flight mass spectrometer, while for MIBI, secondary ions are detected by a magnetic sector or a time-of-flight mass spectrometer. Each heavy metal representing a specific antibody epitope acts as a proxy for cellular protein expression.

Other multiplexed imaging techniques such as CO-Detection by indEXing (CODEX) and tissue-based Cyclic Immunofluorescence (tCyCIF) use fluorescence readouts. CODEX uses antibodies conjugated to short DNA oligonucleotides with iterative cycles of three fluorophore-conjugated complementary oligonucleotides to achieve multiparameter imaging (up to 55 parameters per single cell) [56, 65–67]. tCyCIF uses fluorophore-conjugated antibodies also in an iterative manner but bleaches out the fluorescence between cycles [68]. However, one limitation for all these imaging platforms is detecting low abundance proteins. A multiplex imaging platform, Immuno-SABER, has been designed to overcome this limitation by implementing a signal amplification step with primer exchange reactions [69].

## Carboplatin resistance

### Treating patients with carboplatin-based chemotherapy

Disease management of HGSOC patients progressing after one or more cycles of chemotherapy centers around treating recurrent disease, arising from platinum resistance [3, 70, 71]. Some of the existing treatment options particularly PARPi significantly improve survival, but to date in most cases, none have been curative. Platinum response is defined clinically by the platinum-free interval (PFI), defined as time from the last platinum dose to relapse detection. A PFI of < 1 month classifies a patient as platinum refractory, < 6 month as platinum resistant, 6 month–1 year partially sensitive, and > 1 year as platinum sensitive. While these definitions of PFI act as practical “biomarkers” and are used routinely for guiding treatment, there is no supportive molecular or proteomic information to distinguish between them. After > 3 recurrences, PFI has no predictive or prognostic value. Recently, PARPis were approved in first- and second-line maintenance settings as they significantly increase the PFI [24, 26, 72]. Thus, the use of PFI to stratify patient responses now needs to be modified to include PARPi.

It is unclear if the widely varying duration of PFIs is related to cell autonomous mechanisms or due to influences from the HGSOC iTME. Multiparameter single-cell proteomic technologies are perfectly suited to unraveling the component cell subtypes in order to identify and target resistant cells.

### Carboplatin mechanism of action

Carboplatin is a second-generation platinum anti-cancer agent designed to reduce some of the toxic effects caused by the more chemically reactive cisplatin and is commonly used to treat patients with ovarian cancer. Adequate intracellular levels of carboplatin are key for its genotoxic activity and arise from the balance of drug influx and efflux. These processes are mediated by solute carrier, copper, and multi-drug resistance protein-1 transporters as well as passive uptake into the tumor [73].

Both cisplatin and carboplatin rapidly modify proteins by covalent linkage. With slower kinetics, DNA and RNA are also modified via monoadducts formed by covalent binding to N-7 of guanine followed by either intra- or inter-strand crosslinks and eventually lethal double-strand breaks [74]. These structural distortions activate the DNA damage response (DDR) leading to cell cycle arrest and halting DNA replication and transcription, allowing the cell time to repair the DNA lesions. Failure to do so results in apoptosis or mitotic catastrophe [75, 76].

### Mechanisms of carboplatin resistance

Understanding the mechanistic differences between primary and acquired platinum resistance is key for developing therapeutic agents to achieve curative outcomes. Both types of resistance may be related to specific mutational processes that drive clonal evolution [77–79]. At the genomic level, amplification of *CCNE1* and FBI are associated with primary carboplatin resistance whereas acquired carboplatin resistance is linked to multiple independent reversions in *BRCA1* and *BRCA2*, loss of *BRCA1* promoter methylation, and overexpression of the MDR1 drug efflux pump [6, 7, 80, 81].

Recent studies refined the genomic stratification of HGSOC through the definition of copy number signatures which were shown to be associated with mutational processes such as HRD, FBI, and CCNE1 amplification, and Ras and PI3K-Akt signaling [6, 14]. Of the seven identified signatures, one was correlated with platinum-resistant relapse. Furthermore, individual tumors were shown to harbor one or more copy number signatures suggesting co-evolution of multiple mutational processes that could account for the challenges in overcoming carboplatin resistance.

Both bulk and single-cell transcriptomic studies have provided valuable information about platinum resistance and its correlation with different cellular states, such as inflammatory, immunoreactive, and mesenchymal [53, 54, 82]. Importantly, these studies have provided evidence that the HGSOC iTME plays a role in response to DNA damaging agents. However, it is yet to be determined how different genomic and/or transcriptomic signatures converge upon a more limited set of cellular neighborhoods (see “Multiplex imaging” section) with targetable intracellular signaling pathways.

Carboplatin resistance results from diverse cellular mechanisms which operate in different combinations within and across tumors. Below, we highlight the major mechanisms that have been reported and propose the integration of multiparameter single-cell proteomic data (CyTOF and multiplex imaging) with genomic and transcriptomic datasets to gain a more comprehensive characterization of the tumor microenvironment that distinguishes platinum-sensitive and platinum-resistant tumors.

The efficacy of carboplatin-mediated anti-tumor activity is regulated by its intra-nuclear levels. One mechanism of acquired carboplatin resistance is through decreased intra-nuclear levels arising from changes in expression levels of influx and efflux transporters and/or carboplatin deactivation through adduct formation with glutathione, which is frequently elevated in tumor cells [83].

In response to DNA damage, the DDR, in concert with cell cycle regulation, coordinates a complex network of proteins to maintain the integrity of the genome [75, 84, 85]. Specifically, mutations in homologous recombination (HR) repair genes such as *BRCA1*/*BRCA2*, and others critical for repairing double-strand DNA breaks, increase the lifetime risk of developing ovarian cancer. However, these same mutations sensitize cells to carboplatin [70]. A major mechanism for acquired resistance to carboplatin, shown both in vitro and in vivo, is restoration of HR repair function usually through secondary mutations in HR genes and less frequently through epigenetic changes and increased expression of BRCA1 and other HR proteins [7, 70, 86, 87]. Additionally, acquired restoration of HR has been reported due to loss of 53BP1 with consequent attenuation of non-homologous end-joining, a second major repair pathway for double-strand breaks [88].

**Fig. 2:**
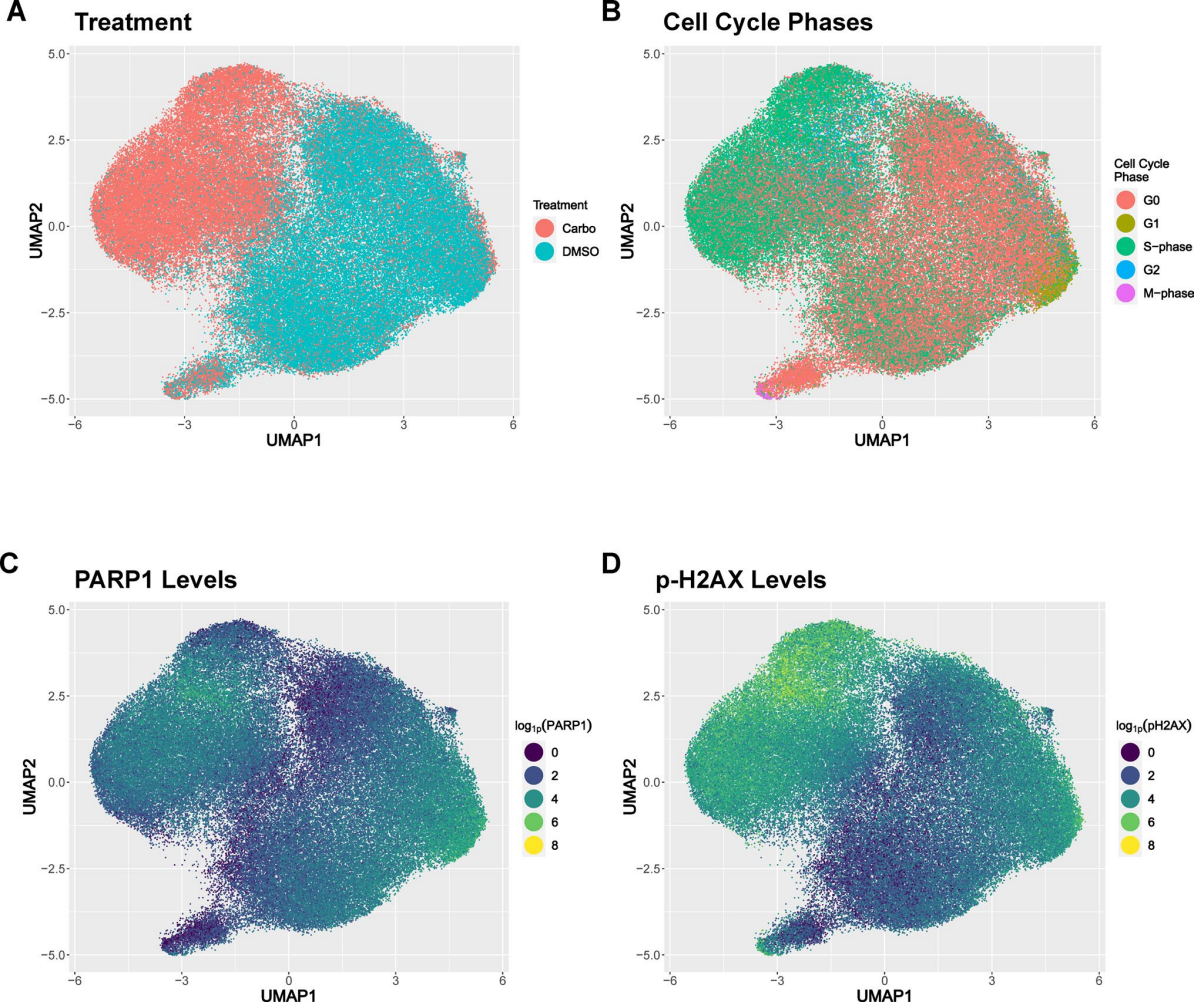
CyTOF reveals heterogeneity of PARP1 and pH2AX protein levels in OVCAR3 cells. OVCAR3 cells were treated with carboplatin (8 µM) or DMSO (control) for 48 h. Cells were stained with CyTOF antibodies against DDR and cell cycle proteins. After normalization, the two CyTOF datasets were concatenated and analyzed by UMAP embedding, a dimensionality reduction algorithm using DDR and cell cycle protein levels. UMAP colored to depict **A** treatment (carboplatin or control), **B** cell cycle, **C** PARP1 expression, **D** pH2AX expression (a surrogate marker for HRD)

The complexity of the DDR was uncovered through numerous genetic, biochemical, and proteomic studies but few have investigated the proteomic response with single-cell resolution [84, 89–92]. Our CyTOF analysis has shown how levels of two key DDR proteins pH2AX and PARP1 differ across single cells within cell cycle phases suggesting an as yet unrecognized level of regulation (*Manuscript in Preparation* and Fig. 2).

Other, less clearly defined mechanisms of platinum resistance in ovarian tumors include the presence of potential cancer stem cells, hypoxia, decreased glucose metabolism, and remodeling of the extracellular matrix [70, 71, 93, 94]. Additionally, proteomic analysis of untreated and recurrent matched patient samples showed increased expression of STAT5 and NFkB in the latter recurrent tumors [95]. Both these transcription factors regulate the anti-apoptotic protein Bcl-Xl.

### PARP and PARPi

PARP1 is rapidly recruited to sites of single-strand DNA breaks where it catalyzes the synthesis of protein-conjugated polymers of ADP-ribose (poly (ADP ribose (PAR)) to itself (auto-parylation) and other proteins orchestrating a DNA repair complex [23]. PARPi inhibits this repair process leading to an accumulation of double strand breaks. When the HR pathway is deficient, double-stand breaks induced by PARPi are unrepaired inducing cell death by synthetic lethality [22, 23]. In HGSOC patients who initially respond to carboplatin, PARPi have shown an impressive survival benefit for women with advanced HGSOC that extends beyond their 2-year administration in a maintenance setting [24, 26, 27, 72, 94]. However, even with this extended PFI, most patients eventually develop resistance to PARPi and as with platinum resistance, subsequent survival of patients with recurrent disease is poor.

Within the complexity of PARPi resistance mechanisms, there is significant overlap with those of platinum resistance especially in tumors where HR is restored. Nevertheless, there are several notable distinctions. While intranuclear levels for both drugs decrease with the increase in expression levels of the MRP-1 efflux transporter, there is as yet no evidence to suggest that PARPi levels are regulated by copper transporters.

### Understanding carboplatin resistance mechanisms within the context of the iTME

So far, our discussion about carboplatin resistance has focused on cell autonomous mechanisms. However, multiple pre-clinical mechanistic studies in a variety of malignancies (including HGSOC) have established the presence of DNA damage-induced innate and adaptive immune activation [96–99]. This necessarily implicates crosstalk between iTME in responsiveness and resistance to DNA damaging therapeutic agents [54, 100–102]. For HGSOC, several studies provided evidence for both innate and adaptive immunity in patient samples using scRNA-seq, multiplex cellular imaging, IHC, and flow cytometry [103–107]. However, characterizing samples biopsied at different times (before or during carboplatin-based treatment) and small sample sizes makes it difficult to derive clinically meaningful conclusions. Characterizing molecularly characterized samples from large clinical trials using multiplex single-cell proteomic technologies holds great potential to further the understanding of DNA-damage-mediated immunity and its role in carboplatin resistance.

## Immunotherapy

### Understanding the HGSOC iTME

The rationale for using immunotherapy stems from the hypothesis that an exhausted host immune system can be reinvigorated to restore its anti-tumor function. Immune checkpoint blockade (ICB), a commonly used and clinically approved modality, targets markers of exhausted T cells [108, 109]. The state of T cell exhaustion occurs after a series of stepwise anti-tumor events against neoantigens expressed by tumor cells or released after their death [110]. To date, ICB using antibodies against PD-1, CTLA4, and PD-L1 has produced robust and durable responses in several advanced solid cancers including melanoma, kidney, bladder, and non-small-cell lung tumors. However, many patients do not experience complete response exhibiting either primary or acquired resistance to treatment [111].

For HGSOC, large, randomized trials with ICB alone or combined with first-line standard chemotherapy and/or bevacizumab have demonstrated minimal clinical benefit across molecularly selected patient sub-groups (germline or somatic BRCA1/2 mutations, HRD, PD-L1 status, or high tumor mutational burden) [112–114]. This was particularly disappointing given that HGSOC tumors harbor exhausted intra-tumoral T cells as well as macrophages and tumor cells that express PD-L1 [112, 115]. Furthermore, combining ICB with cytotoxic agents, to potentially increase neoantigen load also failed to have an impact [112].

**Fig. 3:**
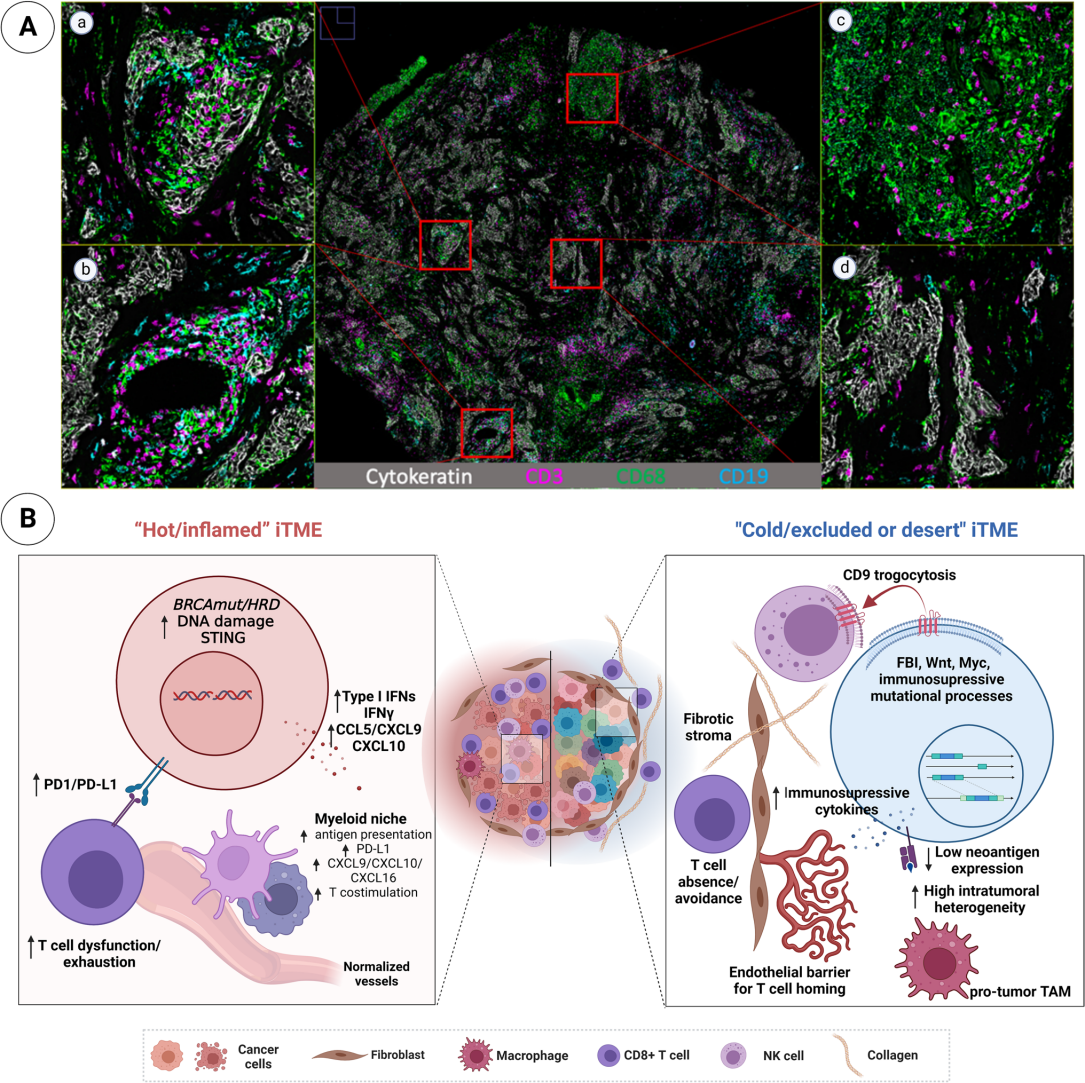
Multiplex image showing micro-heterogeneity of an HGSOC tumor sample after NACT. This tumor exemplifies the existence of several iTMEs within a single tumor sample. Enlarged images reveal different iTMEs. (A-a) “Hot/inflamed” iTME: tumor cells (cytokeratin) infiltrated with T cells (CD3), macrophages (CD68), and B cells (CD19). (A-b) Mixed iTMEs: this part of the tumor shows areas that are “hot/inflamed”’ and “cold/excluded or desert”. (A-c) “Cold” iTME: abundance of macrophages with spatially distinct T cells with absence of tumor cells. (A-d) “Cold/excluded” iTME: tumor-rich region with non-infiltrating immune cells. (B) Schematic showing mechanisms reported to shape the HGSOC iTME. TAM, tumor-associated macrophage; IFNs, interferons; FBI, foldback inversions

Two major conclusions can be drawn from these clinical data. First, HGSOC exhibits the phenomenon of “immune privilege” with a very immunosuppressive iTME that prevents an adaptive immune response from occurring and overrides any clinical benefit from reversing T cell exhaustion. The second conclusion is that a cell-by-cell analysis of the HGSOC iTME is necessary to identify immune suppressive mechanisms with the eventual goal of identifying key druggable targets to reverse this state [116] (Fig. 3).

To identify immune cell phenotypes that could potentially override any reversal of ICB immune activation, our recent mass cytometry study characterized natural killer (NK) and intra-tumoral T cell subsets in untreated HGSOC tumors [117]. This study identified an infiltrating NK cell phenotype with decidual-like features (dl-NK cells), expressing CD9, that was positively correlated with tumor mass [117]. In vitro studies showed that NK cells gained CD9 by trogocytosis (Fig. 3). CD9 is a hallmark of decidual NK (d-NK) cells that make up ~ 75% of uterine lymphocytes during the first trimester of pregnancy, conferring a strong immune suppressive phenotype to prevent a mother-to-be from rejecting her hemiallogeneic fetus [118–120]. From an evolutionary standpoint, this is one of the strongest biological mechanisms to promote species survival. By analogy, in HGSOC, infiltrating dl-NK cells co-opt this mechanism to support tumor survival by providing a strong tolerizing environment that is potentially resistant to immunotherapy. Importantly, frequencies of these dl-NK cells varied across tumors, and one could envision these cells as a biomarker where high and low frequencies could stratify non-responders from responders to ICB respectively. However, it is unlikely that one cell type could be a universal biomarker for HGSOC, and other biomarkers are likely to be revealed by further interrogation of the iTME. Thus, four out of ten paired tumor tissue samples from individual patients taken before and after carboplatin-based neoadjuvant chemotherapy (NACT), and before debulking surgery, showed an increase in nectin-4, the ligand for the inhibitory TIGIT receptor, known to be increased in tumors with acquired resistance to chemotherapy [117, 121]. An independent study analyzed tumor samples before and after NACT by RNA sequencing and noted that cytotoxic gene expression patterns for NK and CD8 T cells were increased after NACT [122]. However, to be cytotoxic, NK and CD8 T cells require ligand engagement with their activating receptors. Thus, one avenue for further biomarker investigation is to measure interactions between NK and CD8 T cells with receptor ligands expressed by tumor cells.

### iTME architecture as a determinant of response to immunotherapy

Multiple studies have described the extent of lymphocyte infiltration within tumors as a determinant of tumor progression and response to treatment [123]. For ovarian cancer, the extent of CD3 T cell and CD8 T cell infiltration within tumor islets correlated with more favorable survival [124, 125]. In a subsequent study, HGSOC tumors and ascites with high numbers of immunosuppressive regulatory T cells correlated with reduced survival [126]. More recently, the spatial distribution and frequencies of infiltrating T cell classified tumors as “hot” (highly infiltrated and inflamed), “excluded” (T cells found at periphery of tumor islet in stroma), and “desert” (low T cells frequency or absent) [127, 128]. We show the co-existence of these widely differing immune states within the same HGSOC tumor after NACT (Fig. 3(A)) and summarize the mechanisms by which HGSOC can manifest different immune phenotypes. In our CODEX image, the “cold” immune phenotype encompasses “immune excluded and desert” (Fig. 3(A, B)). Substantial tumor associated macrophages (TAMs) seen in this sample are consistent with the central role played by these immune cells in HGSOC disease progression and chemoresistance. TAMs are comprised of anti-tumorigenic M1 and pro-tumorigenic M2 populations. High tumor frequencies of M2 TAMs have been shown to correlate with poor survival [129, 130].

Although these and other studies provided evidence for the relationship between immune cell infiltration with prognosis and response to immunotherapy, they do not address the complexity of the iTME as a spatially organized assembly of phenotypically distinct sub-populations of tumor, immune, stromal, and angiogenic cells with extracellular matrix components. As with healthy tissues, frequencies of specific cell phenotypes and their proximal and distal spatial organization play a critical role in tissue/tumor function, which in the case of the latter relates to prognosis and response to immunotherapy [131]. The availability of multiplex imaging platforms has enabled the identification of spatial biomarkers, derived from topological differences between cell–cell interactions, that stratified clinical outcomes for patients with colorectal and breast cancer, cutaneous squamous cell carcinoma, and T cell cutaneous lymphoma [56, 61, 64, 132]. Recent studies have shown the value of including spatial interactions between tumor cell types to develop biomarkers with the potential to predict response of melanoma patients to anti-PD-1 [133–135]. At present, there are no approved companion diagnostics for this clinical setting. However, in an independent meta-study for predicting response to anti-PD-1 /PD-L1, spatial biomarkers outperformed more traditional methods of patient stratification (such as gene expression profiling, tumor mutational burden assessment, and PD-L1 IHC) [134, 135].

### Characterizing the HGSOC iTME through the lens of transcriptomics

A wealth of transcriptomic data from both bulk and single-cell sequencing have further highlighted the immense heterogeneity of the HGSOC iTME [54, 82, 136, 137]. Moreover, unlike breast cancer, where gene expression-based predictors are now used routinely to guide therapeutics, this is not the case for HGSOC [138]. Nevertheless, the insight gained from transcriptomic profiling of HGSOC serves as an important foundation for proteomic analysis.

A recent scRNA-seq study of ascites and solid tumors from advanced-stage HGSOC showed extensive heterogeneity among tumor cell types across samples [53]. The results should be interpreted with caution as samples were not stratified according to treatment with chemotherapy (they were untreated, on initial treatment or on-treatment for recurrent HGSOC) and furthermore were not typed for HRD [53]. However, subpopulations of cancer-associated fibroblasts, macrophages, and tumor cells expressing the MHC class II program were shared across samples. The latter may be an indication of increased intra-tumoral lymphocytes and potentially could predict response to immunotherapy [139]. These results warrant follow-up with single-cell proteomic technologies.

Understanding the underlying molecular mechanisms that shape the different T cell infiltration patterns (“hot/inflamed,” “cold/excluded or desert”) potentially holds key information for identifying new therapeutic targets. Additionally, more reliable biomarkers may be found that improve the selection of patients eligible for immunotherapy both before and after chemotherapy. A recent study, rather than using genomic or survival data, pre-selected HGSOC tumors as immune “hot,” “excluded,” or “cold” using CD8 IHC and bulk RNA-seq [140]. Five tumors representing each subgroup were dissociated into single cells and fluorescence-activated cell sorting was used to isolate tumor, immune, and stromal populations. Each population was subsequently profiled by scRNA-seq. “Hot” and “cold” iTMEs differed markedly both in their tumor cell autonomous and non-autonomous transcriptional programs. Key findings from this study revealed that tumor cell autonomous transcriptional programs in “hot” and “excluded” tumors were enriched for oxidative pathway and antigen-presenting machinery gene sets, whereas “cold” tumors were enriched for EMT and angiogenesis gene sets. Transcriptional programs for T cells were consistent with “hot” tumors having an enrichment of advanced dysfunctional CD8-granzyme B T cells while “excluded” tumors were enriched with pre-dysfunctional, effector memory CD8-granzyme K T cells [140, 141]. TAMs also differed between the tumor immune phenotypes. Most notably myeloid-derived suppressor cells present in immune “cold” tumors were absent in immune “hot” and “excluded” tumors. Furthermore, specific chemokine receptor ligand interactions (e.g., CXCR6/CXCL16) were identified suggesting their roles in regulating the observed T cell infiltration patterns as a result of crosstalk between tumor, immune, and stromal cell compartments.

Through transcriptomic profiling of HGSOC, this study provided key mechanistic insight about their different iTMEs. However, it did not account for micro-heterogeneity within HGSOC tumor tissue. Although tumor samples were selected as immune “hot,” “excluded,” and “cold,” they are likely to be comprised of a mix of iTME phenotypes which would have been missed (Fig. 3) [122, 142–144]. Since, as previously described, the iTME acts through coordinated cell–cell interactions, and not through random interactions, spatial cellular arrangements, derived from multiplex proteomic imaging technologies, are expected to directly address HGSOC micro-heterogeneity and generate data to improve predictive and prognostic biomarkers for this and other malignancies [56, 61, 145].

### Imaging the HGSOC iTME

Multiplex imaging of the HGSOC iTME is now emerging as a key technology for understanding response to therapies and immune escape [61, 146]. In a recent single-arm non-randomized phase I/II trial study (TOPACIO), patients, mostly with platinum-resistant HGSOC and irrespective of *BRCA1* or *BRCA2* status, were treated with a combination of ICB (pembrolizumab) with a PARPi (niraparib) [146, 147]. The rationale for this combination was based on limited data showing that tumors with HRD have both a greater neoantigen load and a cytotoxic T cell infiltrate and that PARPi would bolster these parameters [148]. Furthermore, two preclinical studies, in a setting of either mutated or wild-type *BRCA* respectively, showed that PARPi treatment led to the accumulation of cytosolic double-strand DNA fragments because of unrepaired DNA lesions [106, 149]. The latter is an activator of the cyclic GMP–AMP synthase (cGAS)–stimulator of interferon genes (STING) pathway, a key mediator of innate immunity. Moreover, PARPi gave rise to increased frequencies of intra-tumoral CD8 and CD4 T cells [106, 112, 149–151].

In the TOPACIO clinical trial, a small number of patients exhibited a complete or partial response to ICB plus PARPi [147]. Consequently, banked tumor samples from the study were retrospectively characterized by genomic and transcriptomic profiling and tCyCIF multiplex imaging. More favorable outcomes were associated with HRD and/or a transcriptomic score representing interferon primed exhausted CD8 T cells. Spatial imaging analysis of two extreme responders showed different cellular interactions of exhausted CD8 T cells with either macrophages expressing PD-L1 or tumor cells with gene amplification of PD-L1 and PD-L2 (Fig. 3). The results of this non-randomized trial should be viewed with caution as they were all banked before the start of the ICB-PARPi trial. They were taken from patients, either untreated or while on chemotherapy, and represented a single metastatic site per patient. Finally, the trial was single-arm combination study making it impossible to distinguish the effects of each agent.

Nevertheless, the results from the trial are consistent with the association of HRD, including *BRCA1/2* mutations, with a distinct, more immunogenic iTME. Furthermore, although *BRCA1/2* status is a biomarker for more favorable progression-free survival (PFS), that is not always the case as a small sub-group of patients with *BRCA1/2* mutations have short PFS, even when treated with maintenance PARPi [152, 153]. Thus, spatial parameters derived from immune cell arrangements in the iTME may provide more reliable biomarkers for identifying sub-groups of patients who would benefit from immunotherapy. Two groups used multiplex imaging to compare the iTME of tumors harboring wild-type or *BRCA1/2-*mutated genes [154, 155]. Both studies showed that the iTME in a *BRCA1/2*-mutated setting had greater frequencies of CD8 T cells, macrophages, and PD-L1-expressing tumor cells and fibroblasts than in the *BRCA1/2* wild-type setting. The studies described above highlighted the need for additional mechanistic preclinical studies and large-scale randomized clinical trials designed to include tumor biopsies before and during treatment with ICB in combination with chemotherapy, PARPi, and other agents such as STING agonists.

## HGSOC heterogeneity

### Incorporating heterogeneity into biomarker development for HGSOC

Advanced-stage HGSOC typically presents with multiple metastatic lesions in the abdominal cavity. Genomic and iTME heterogeneity of HGSOC is present within a given lesion, between lesions in the same patient or across lesions in different patients [77, 79, 122, 156]. A recent study integrated genomic, transcriptomic, B cell receptor, and T cell receptor sequencing with immunohistochemistry to comprehensively profile 212 multiregional samples at diagnosis from a 36-patient cohort [144]. The results confirmed the HGSOC heterogeneity with intra-patient, site-specific variations in levels of “immune privilege,” and patterns of immune infiltration that correlated with specific mutational sub-groups. One result in particular, from this study, highlights the need for multimodal biomarker analysis. High immune activity was observed in two mutational subgroups of tumors. The subgroup with *BRCA1/2* mutations had favorable outcomes whereas tumors with FBIs had poor outcomes. It is intriguing to speculate whether FBI tumors harbor greater frequencies of dl-NK cells [117].

The recurring theme from this review is that one HGSOC biopsy taken at one anatomical site before treatment is insufficient to capture the overall disease heterogeneity within an individual patient. Furthermore, a single immune privileged microenvironment could be sufficient to nullify any reversal of immune exhaustion by ICB at other sites. An additional clinical challenge is that over time and in response to treatment HGSOC undergoes profound changes brought about by clonal evolution [79, 122, 142]. Together, these data highlight the importance of analyzing HGSOC biopsies over time. The data also support developing multimodal biomarkers using datasets generated from orthogonal technologies [10]. A potential example of a multimodal biomarker would be the incorporation of genomics with multiplex cellular imaging revealing spatial information about tumor–immune cell interactions, and expression of receptor ligands for T and NK cells to track disease progression and/or determine if a patient is a candidate for immunotherapy [117, 122, 144]. The data also highlight the need to convert immune privileged sites into sites that are immunogenic, an area of intense investigation [157, 158].

### Incorporating radiomics into the development of multimodal biomarkers

Ongoing efforts are being evaluated for incorporating radiomics in the development of multimodal biomarkers for HGSOC, and a variety of other malignancies [159–163]. Radiomics quantifies images, usually derived from computed tomography (CT) that are routinely used to monitor disease progression [164]. Unlike tumor biopsies, CT scans are non-invasive, not limited to one anatomical site, and monitor changes in tumor burden over time. However, radiomics does not capture the cellular heterogeneity of the iTME. Radiomics uses artificial intelligence analysis methods to extract textural features directly from CT images to derive “habitats.” Combining the complementary information from radiomics, clinical parameters, genomics, and histopathology into a multimodal biomarker was recently shown to be a more accurate measure for predicting PFS and platinum resistance than conventional or average radiomic measurements [160, 161].

## Future directions and challenges

**Fig. 4:**
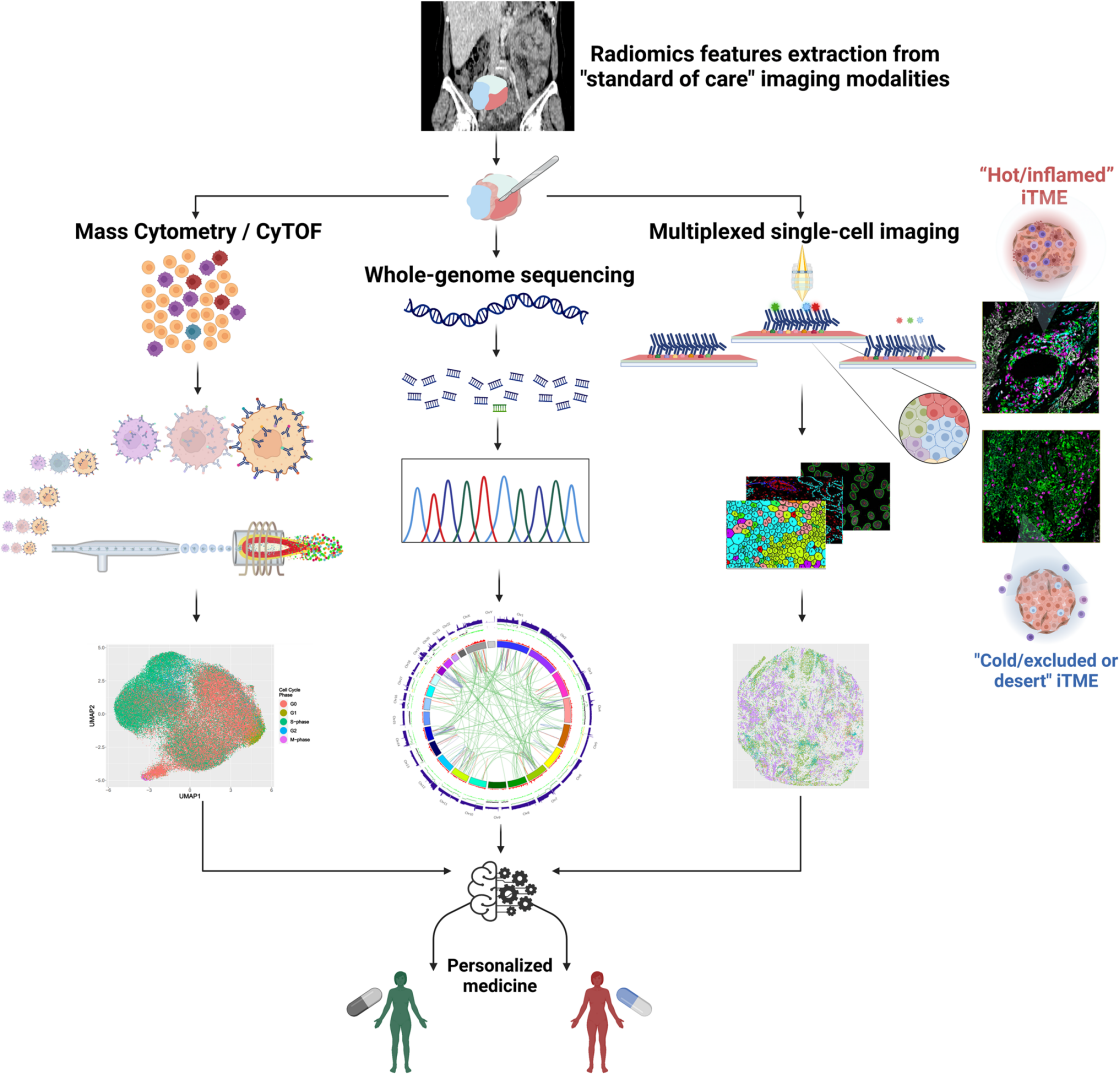
Multimodal biomarkers—personalized medicine for women with HGSOC. A multimodal biomarker comprised of data from radiomics, genomics, CyTOF, and multiplex imaging. Fluorescence-based multiplex imaging shown as a representative imaging platform. Circos plot of whole genome sequencing of DNA from HGSOC patient with *CCNE1* amplification and HRP

Deconvolution of the HGSOC iTME will be essential to understand and overcome platinum and PARPi resistance as well as to reveal new therapeutic avenues to reinvigorate the anti-tumor immune response. Intricate genomic and biological processes associated with complex cell–cell interactions produce extreme heterogeneity of HGSOC warranting implementation of a multimodal approach for its understanding (Fig. 4). Published studies with multimodal biomarkers that are composites of readouts from genomics, radiomics, transcriptomics, and immunohistochemistry are more reliable than unimodal biomarkers. We propose that the accuracy of multimodal biomarkers for HGSOC can be improved further by incorporating a metric(s) derived from CyTOF and/or multiplex single-cell imaging data. Integrating cell types and their neighborhood interactions within the iTME (micro-scale) with “habitat” heterogeneity (radiomics at meso-scale) within the context of genomic heterogeneity will provide an unprecedented level of clinically actionable information. To validate this multimodal approach, it will be essential for clinical trials to have built-in infrastructure to access highly annotated patient samples before, during, and after treatments.

## Data Availability

The datasets generated during and/or analysed during the current study are available from the corresponding author on reasonable request.
